# The economic burden of knee and hip osteoarthritis: absenteeism and costs in the Dutch workforce

**DOI:** 10.1186/s12891-022-05306-9

**Published:** 2022-04-18

**Authors:** Marrit Hardenberg, Erwin M. Speklé, Pieter Coenen, Iris M. Brus, P. Paul F. M. Kuijer

**Affiliations:** 1 Department of Public and Occupational Health, Amsterdam UMC, Vrije Universiteit Amsterdam, Amsterdam Public Health Research Institute, van der Boechorststraat 7, 1081 BT Amsterdam, The Netherlands; 2 Arbo Unie, Occupational Health Service, Utrecht, The Netherlands; 3 Department of Public and Occupational Health, Amsterdam UMC, University of Amsterdam, Amsterdam Movement Sciences, Amsterdam, The Netherlands

**Keywords:** Cost of illness, Health economics, Health Expenditures, Lower-limb osteoarthritis

## Abstract

**Background:**

This study aimed to quantify the absenteeism costs of knee and hip osteoarthritis in the Netherlands for the Dutch workforce and specific groups of workers.

**Methods:**

We used a longitudinal, dynamic database from a large occupational health service in which occupational physicians register information about personal information and sick leave of workers with the diagnosis of knee- and/or hip osteoarthritis. We included all employees aged 15 to 75 years performing paid work and diagnosed with knee and/or hip osteoarthritis. Costs were calculated annually and per episode for different subgroups from an employer’s perspective using the Human Capital Approach. In the Netherlands, the employer has to pay 70% of the employee's wage out of pocket for the first two years of sick leave and also for the occupational health care. In this way, employers receive information about the costs of workers on sick leave due to knee or hip osteoarthritis. This might stimulate investments in targeted prevention and work-directed care.

**Results:**

For the period 2015–2017, 1399 workers fulfilled the inclusion criteria. An average sick leave episode of knee osteoarthritis had a duration of 186 calendar days and was associated with €15,550 in costs. For hip osteoarthritis these data were 159 calendar days and €12,482 in costs. These costs are particularly high among male workers and workers with a higher number of weekly working hours. The average annual costs for the Dutch workforce due to sick leave for knee and hip osteoarthritis were €26.9 million and €13.8 million, respectively. Sick leave costs decreased for hip and not for knee osteoarthritis during 2015–2017.

**Conclusions:**

Annual sick leave costs due to knee and hip osteoarthritis are about €40 million for the Dutch workforce and approximately twice as high for knee compared to hip osteoarthritis. Average costs per sick leave episode are particularly high among male workers and workers with a higher number of weekly working hours.

**Supplementary Information:**

The online version contains supplementary material available at 10.1186/s12891-022-05306-9.

## Background

Osteoarthritis (OA) puts a high burden on society. Direct medical costs for OA in the Netherlands were estimated in 2017 at €1.2 billion (€488.2 million and €433.4 million for knee and hip OA respectively) [1]. This corresponds to 18% of the costs for diseases of the musculoskeletal system, and 1% of the total annual health care costs [1]. However, the economic burden is not limited to these direct medical costs and also consists of indirect costs, that are generally based on productivity loss due to sick leave (absenteeism) and sickness while at work (presenteeism) [2, 3]. Research from the Netherlands in 2012 showed that indirect costs accounted for approximately 83% of the total economic burden of OA [4].

Although OA is one of the leading causes of sick leave in the Netherlands, recent evidence on the costs of sick leave is lacking [5]. The most recent report on the costs of OA in the Netherlands dates back to 2012, in which the costs of knee-related productivity and medical costs in knee OA patients with paid employment were estimated [4]. The lack of evidence on indirect costs possibly prevails because there is debate regarding the best method to measure productivity costs (e.g. cost calculation approach, and handling of skewed and missing data), and because data on productivity are often difficult to obtain [3, 6, 7]. In the Netherlands no reliable national registries for sick leave and absenteeism data are available. Therefore, these data must be obtained through surveys and occupational health services [6]. The corresponding methodological issues are reflected in the lack of studies on productivity costs, as only 10% of cost-effectiveness studies include these costs [8–10]. Consequently, it is unknown how productivity costs of knee- and hip OA have developed over recent years and if these costs differ for specific subgroups of workers.

This gap in the literature is unfortunate because data on sick leave due to OA are necessary for employers, and occupational health professionals in the prioritization and development of interventions to prevent and manage these work-related diseases [5]. In the Netherlands, employers have to pay out of pocket for the services provided by these professionals and also 70% of the employee's wage for the first two years of sick leave according to the Dutch Gatekeeper Improvement Act. Especially data on subgroups could help target these interventions. This study aimed to quantify the sick leave costs of knee and hip OA in the Netherlands for the total Dutch workforce and specific groups of workers.

## Methods

### Study design, setting, and participants

Data for this cohort study were obtained through one of the largest occupational health services, Arbo Unie. They maintain a longitudinal, dynamic database from approximately 1.2 million workers from several occupational sectors throughout the Netherlands. The database was composed from records of occupational physicians in the period 2013 to 2020. These physicians guided workers who were, in general, on sick leave for longer than a week or have high risk of long-term sick leave. The database is hosted by a third trusted party and access to the database can be requested from Arbo Unie via an email to the authors. The request will then be forwarded to the governance body that controls access to the database. If the request fulfils the ethical and research criteria the data will be released. Please note, that there is a fee to pay for the costs that are being charged by the third trusted party for the release of the data.

The inclusion criteria were that data were used from all workers that visited an occupational physician and were diagnosed by the occupational physician with knee and/or hip OA. In addition, this diagnosis was established as the main reason of sick leave. We included employees that fit the definition of ´worker´ according to Statistics Netherlands: people aged 15 to 75 years and performing paid work [11]. Workers with a contract < 4 h a week were excluded, because of the high probability that these workers were on-call, weekend and/or evening workers, and the actual number of working hours could not be estimated for these workers. The database was initially composed for care-related purposes and not for research. Therefore, no informed consents were obtained from the workers in our database. Due to the magnitude of the database and since data were fully anonymized, the Medical Ethical Committee of the Amsterdam UMC (location VUmc) granted permission for the use of this database for research purposes and that no post-hoc informed consent were required obtained (METc reference no. 2020.104). Otherwise, study procedures were performed in line with the ethical standards of this local ethics committee and with the Helsinki Declaration of 1975, as revised in 2000.

### Variables

Occupational physicians recorded personal information (i.e. birth year, sex, and weekly working hours) and information related to sick leave, like diagnosis using the Classification system for Occupational and Social insurance physicians (CAS), with L642 for knee OA (ICD code M17) and L641 for hip OA (ICD code M16), sickness absence duration, and recovery percentage (0% = fully absent, 100% = fully working according to contract time).

Age was categorized into < 45, 45–49, 50–54, 55–59, 60–64, and ≥ 65 years age groups. The first group consisted of all workers < 45 years because the prevalence of OA in younger people is low [12, 13]. In the last group workers above the age of 64 years were grouped together as this captures workers from pensionable age in the Netherlands [14]. Working hours were categorized into < 20, 20–24, 25–29, 30–34, 35–39, and ≥ 40 h/week groups. The number of sick leave episodes was dichotomized to the first sick leave episode versus every following sick leave episode.

### Cost measurement

Costs were calculated from an employer´s perspective using the Human Capital Approach (HCA). This perspective is chosen because employers have to pay for the prevention and care provided by occupational health services. In addition, the employer has to pay 70% of the employee's wage for the first two years of sick leave based on the Dutch Gatekeeper Improvement Act. If occupational health services have data to show employers what costs might be prevented and for whom, employers might be more willing to invest in targeted prevention and occupational health care. Here, the sick leave hours and the productivity costs were calculated for every sick leave episode. Episodes with less than 28 calendar days in between were considered one episode, using the definition of a sick leave episode from the Dutch employee insurance agency [15]. Sick leave hours per sick leave episode were calculated using sick leave days and contractual working hours from the Arbo Unie database, which were adjusted by the recovery percentage. Productivity costs were extracted from (age and sex-dependent; see Supplementary file 1) average national gross wages that were multiplied by 0.7 because as said, in the Netherlands, an employer is legally required to minimally pay 70% of the worker's wage during the first two years of sick leave based on the Dutch Gatekeeper Improvement Act. After that, the worker is assumed to have a permanent work disability, can be declared unfit for work and no additional sick leave costs are generated for the employer [15]. Therefore, a maximum 2 years of sick leave was adopted.

### Statistical methods

Total annual sick leave days and total annual costs were calculated for knee and hip OA. These variables were then extrapolated from the study sample to the Dutch working population under the assumption that the database of Arbo Unie covered 14% of the working population (1.2 million workers). The Dutch workforce included 8.6 million people in 2017.

To assess differences in sick leave costs between certain subgroups, costs were calculated per sick leave episode and a univariate linear regression analysis was performed for each subgroup (i.e. sex, age, working hours/week, and number of sick leave episodes), after which also a multivariate linear regression analysis was performed. In the multivariate linear regression analysis, sex, age, working hours/week, and number of sick leave episodes were included and the Beta (B) and the corresponding 95% confidence intervals (95% CI) of each variable were calculated adjusted for the other three variables. Due to the right-skewed nature of cost data and the presence of heteroskedasticity, all analyses were performed using bias-corrected and accelerated wild bootstrapping (5000 samples) [16, 17]. All analyses were performed using SPSS version 26.

**Fig. 1:**
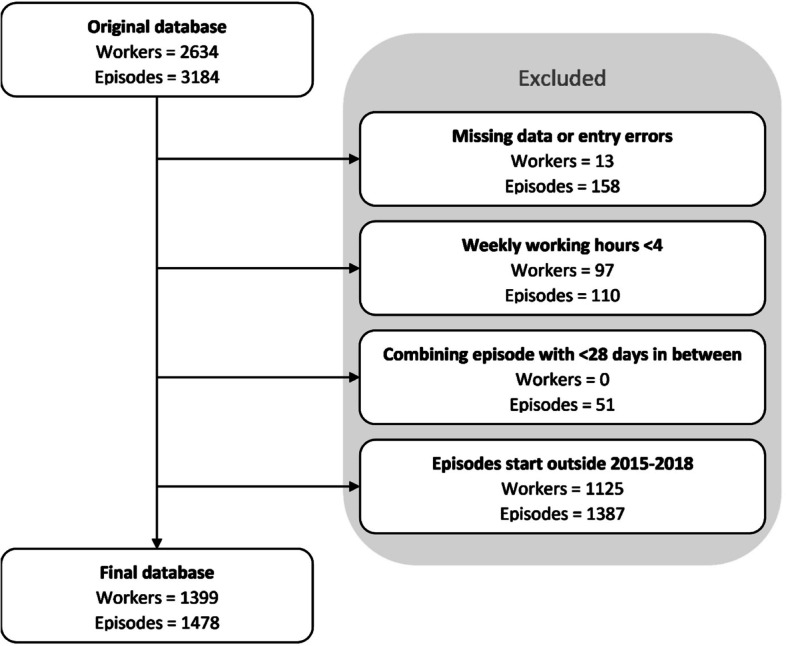
Flowchart depicting the data selection procedures of this study

Only complete cases were analyzed. Workers with missing or unrealistic data, like an end date before a start date, were excluded (Fig. 1). Workers with a start date before 2015 or after 2018 were excluded since a sick leave episode can last up to 2 years due to the Dutch law. Lastly, costs could not be calculated for sick leave episodes that started in 2018 and ended after 2019 because the average national gross wages of 2020 were not available yet. This means average costs per episode were estimated for episodes with a start date in 2015–2018 and an end date at the latest in 2019, and annual costs were estimated over the years 2015–2017.

A sensitivity analysis was performed by using the Friction Cost Approach (FCA) rather than the HCA. According to the FCA, productivity costs only occur during the period necessary to fill the vacancy that has arisen due to sick leave, i.e. the friction period [18]. Therefore, instead of using a maximum of 2 years of sick leave, a friction period was used to which the number of sick leave days were limited. The friction period was estimated per year (see Supplementary file 2), according to the guidelines of the Dutch Healthcare Institute, with data from Statistics Netherlands [19] with the formula:

$$\mathrm{Friction period}=365 /\frac{\mathrm{Filled vacancies}}{\mathrm{Outstanding vacancies}}+4\mathrm{ weeks}$$

## Results

### Participants

Most workers were male (57%) (Table 1). The number of workers in the dataset increased with each age group with a sharp drop after reaching the age of 65. In the period between 2013 and 2019, the retirement age increased from 65 years and 1 months to 66 years and 4 months in the Netherlands. Most people worked 35–48 h/week. When stratified by sex, males worked a median of 38.0 (IQR 4) hours/week while the working hours for females differed more across participants; females with knee OA worked a median of 24.0 (IQR 15) hours/week and those with hip OA 26.5 (IQR 16) hours/week. Only a few workers (4% and 5% for knee and hip OA, respectively) experienced more than 1 sick leave episode during the examined time span.

**Table 1:** Characteristics of workers on sick leave due to knee and hip osteoarthritis. Number of participants (n) and their % of the total sample are shown

	**Knee osteoarthritis [n(%)]**	**Hip osteoarthritis [n(%)]**
**Workers**	870 (100)	529 (100)
**Sex**		
Male	523 (60.1)	274 (51.8)
Female	347 (39.9)	255 (48.2)
**Age**		
< 45	42 (4.8)	33 (6.2)
45–49	69 (7.9)	53 (10.0)
50–54	126 (14.5)	86 (16.3)
55–59	277 (31.8)	142 (26.8)
60–64	311 (35.7)	196 (37.1)
≥ 65	45 (5.2)	19 (3.6)
**Working hours**		
< 20	143 (16.4)	77 (14.6)
20–24	80 (9.2)	64 (12.1)
25–29	56 (6.4)	44 (8.3)
30–34	97 (11.1)	55 (10.4)
35–39	242 (27.8)	160 (30.2)
≥ 40	252 (29.0)	129 (24.4)
**Number of sick leave episodes**		
1	823 (94.6)	506 (95.7)
> 1	47 (5.4)	23 (4.3)

Annual sick leave costs.

**Fig. 2:**
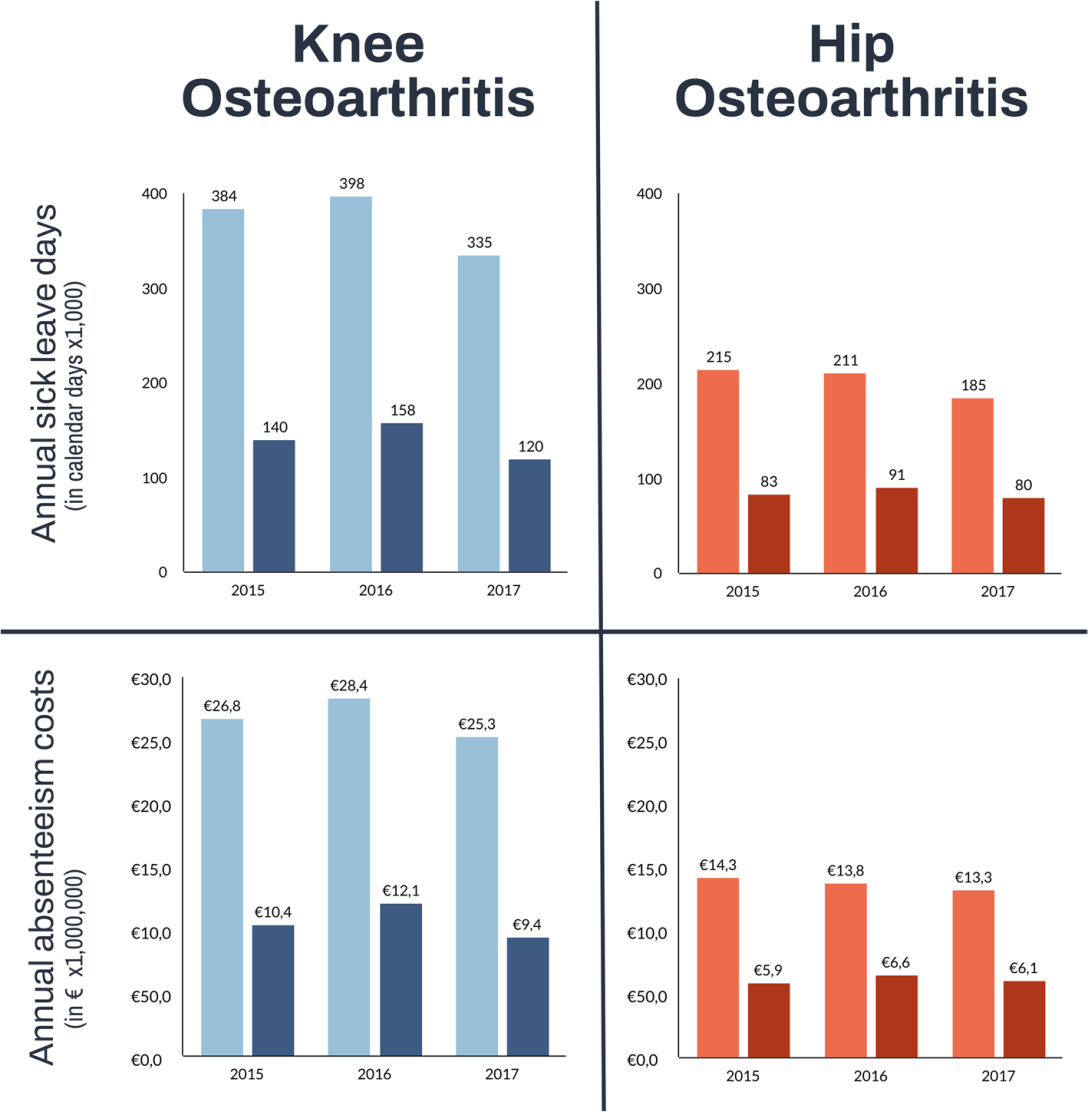
Annual sick leave days (upper panel) and annual absenteeism (lower panel) costs of workers with knee (in blue) and hip osteoarthritis (in red) who consulted an occupational physician extrapolated to the Dutch working population. Dark colours = Human Capital Approach, Light colours = Friction Cost Approach

Annual sick leave days and sick leave costs, calculated from the employer’s perspective using the HCA and extrapolated to the Dutch workforce, are shown in Fig. 2. In 2015–2017, an annual average of 372,099 knee OA associated sick leave days and 203,523 hip OA associated sick leave days were registered. The extrapolated sick leave corresponded with average annual costs of €26.9 million (knee) and €13.8 million (hip). We observed a decline in sick leave costs for hip OA over time, from €14.3 million (2015) to €13.3 million (2017). Linear regression analysis (Table 2), confirmed these results with a statistically significant regression coefficient (Beta) of -67,569 (95% CI -113,658 and -21,480), depicting the annual decrease in costs. For knee OA, no such significant decrease or increase could be discerned given the 95% CI from -2,574,557 to 2,360,146 for the Beta of -107,205 (Table 2).

**Table 2:** Univariate linear regression analysis on annual sick leave costs, depicting an annual change in costs over time

	**Beta**	**95% CI**	***P*-value**
**Human Capital Approach**			
Knee	-107,205	-2,574,557 – 2,360,146	0.679
Hip	-67,569	-113,658 – -21,480	0.034
**Friction Cost Approach**			
Knee	-71,626	-2,382,948 – 2,239,695	0.761
Hip	17,755	-657,374 – 692,884	0.795

### Costs per sick leave episode

The average overall cost per episode of knee OA was €15,550 with an average sick leave duration of 186 days (Supplementary file 3). Analyses showed that females had lower sick leave costs than males, this difference in the univariate model (-€8,796) was attenuated in the multivariate analysis (-€4,360). Also, costs increased with increasing working hours.

The average overall costs per episode of hip OA were €12,482 with an average sick leave duration of 159 days. Females had lower costs per episode than males (-€5,116) despite more sick leave days per episode (173 days compared to 147 days for males). In the multivariate analysis, however, this sex difference attenuated (-€1,875). Only workers aged 65 years and over generated lower costs than the reference group (multivariate: -€6,627). Working hours differed in costs per episode from the reference category with an increase in costs with an increasing number of working hours.

### Sensitivity analyses

In a sensitivity analysis using the FCA we estimated lower costs than when using the HCA (Fig. 2). Average annual sick leave costs due to knee OA were estimated at €10.6 million (40% of the costs calculated using the HCA). For the hip, the average annual amount of sick leave-related costs was €6.2 million (45% of the HCA). We could not find a statistically significant difference in costs over time with the FCA (knee 95%CI = -2,382,948, 2,239,693, hip 95%CI = -657,374, 692,884). Trends between subgroups remained relatively similar between the two approaches (Supplementary file 4).

## Discussion

In the period 2015–2017 in the Netherlands, annual sick leave costs due to knee and hip osteoarthritis are about €40 million for the Dutch workforce and approximately twice as high for knee compared to hip osteoarthritis. Average costs per sick leave episode are €15.550 for knee OA and €12.482 for hip OA. These costs are particularly high among male workers and workers with a higher number of weekly working hours.

### Interpretation of findings

The lack of recent and high-quality research on the topic of indirect costs of OA in combination with methodological heterogeneity makes the comparison with literature difficult [3, 5–7]. Salmon et al. reviewed papers measuring the economic burden of OA worldwide and recalculated them to fit the societal perspective per patient per year [20]. Since we calculated costs per episode from an employer’s perspective, we recalculated our findings to fit the narrative of costs from a societal perspective per patient per year in 2020 euros. The costs from a societal perspective are 8,100 euros (95% CI 7,800–8,300) according the FCA and 13,600 euros (95% CI 13,000–14,100) according the HCA. As it was unclear if certain papers used the HCA or the FCA, both results are displayed in Table 3, which shows a wide variety in OA related sick leave costs between studies and geographical locations.

**Table 3:** Indirect costs of knee- and hip osteoarthritis from societal perspective per patient per year based on our data and as reported in literature. Costs were recalculated to 2020 euros using the consumer price index from each respective country. Blue = current study results. HCA = Human Capital Approach. FCA = Friction Cost Approach.—= Unknown

Author	Country	Participants	Approach	Mean costs (in €, 2020)	95% CI
Present study	Netherlands	1,399	HCA	13,600	13,000 – 14,100
Present study	Netherlands	1,399	FCA	8,100	7,800 – 8,300
Leardini, 2004 [21]	Italy	254	HCA	1,800	1,100 – 2,400
Loza, 2009 [22]	Spain	1,071	-	200	200 – 300
Hermans, 2012 [4]	Netherlands	117	-	10,500	7,900 – 13,100
Rolfson, 2012 [23]	Sweden	2,635	-	6,000	-
Salaru, 2014 [24]	Moldova	256	HCA	300	-
Gupta, 2005 [25]	Canada	1,258	HCA	13,700	12,900 – 14,400
White, 2008 [26]	US	32,043	-	4,700	-
Xie, 2007 [27]	Singapore	80	HCA	1,600	1,500 – 1,700

Our findings are within the same range as those from earlier research in the Netherlands [4]. However, one should keep in mind that the displayed costs from this earlier study are the total indirect costs, often including not only absenteeism but also presenteeism and sometimes lost productivity costs while at home (housework). For example, reported absenteeism costs from Hermans were only €2,775 per patient per year, with additional costs stemming from other indirect sources [4]. Since our findings were based on workers on sick leave who consulted an occupational physician, our mean costs seem high in comparison to this earlier study. This difference in costs might partly be explained by the following two reasons. First, the study of Hermans was conducted among workers with mild to moderate knee OA, while our study did not exclude workers based on the severity of OA. Our sample probably includes more workers with severe OA given their consult with an occupational physician, which might have led to more sick leave days and higher costs. Second, it is known that self-reported sick leave through questionnaires (like Hermans used) leads to lower costs than when sick leave is reported using data from administrative databases (like we did in this study) [28]. In addition, given that only workers on sick leave consulting an occupational physician were included in our study and the fact that in the Netherlands occupational physicians mainly see patients with a longer sick leave period, the actual costs due to sick leave might even be higher. However, given that knee and hip OA are associated with a lower socio-economic position, using average wage costs might result in an overestimation of the costs for the Dutch workforce.

Looking at our findings, there are a few immediately notable results. Firstly, annual costs of knee OA are almost twice the size of costs of hip OA. This difference can be explained by the fact that knee OA, as the most common form of OA, is more prevalent than hip OA and that the average sick leave episode of knee OA is longer than that of hip OA [12]. Also, a decline over the years could be seen for hip OA and not for knee OA. Due to these factors, we recommend occupational health professionals and policymakers to focus interventions for prevention and work-directed care especially on workers with knee OA.

Workers over the age of 65 years seemed to generate lower costs per episode than workers from other age categories, which seems odd since the prevalence of OA increases with age [12]. However, our sample included few workers with OA over the age of 65. A study from Ohio, measuring costs for all kinds of OA among workers, found the same decrease in prevalence after the age of 65 and offered the following two explanations: (1) the ‘healthy worker effect’ where healthy workers stay employed whereas those with health problems or age-related disability leave the workforce or move to less physically demanding jobs and (2) older individuals may retire without surgery [29]. These explanations seem to hold for our findings as well since the pensionable age in the Netherlands in the studied period increased from 65 years and 1 months to 66 years and 4 months and some studies showed that OA is a substantial cause for early retirement [21, 25]. Additional explanations for the lower costs could be that, (3) older individuals have a lower average wage than younger individuals and (4) older individuals may start working fewer hours due to seniority [12]. Nevertheless, in recent years the pensionable age in the Netherlands increased and is expected to increase even further in the coming years [14]. As workers are staying in the workforce longer, the prevalence of OA among workers is very likely to increase and we expect the costs among workers aged older than 65 to rise.

Maybe the most notable findings are that males generate more lost productivity costs than females. If policymakers or employers take on a cost-minimizing strategy, males appear to be the target subgroup. However, females reported more sick leave days than males. Overall females suffer more, longer, and more severely from OA [30, 31]. Therefore, the lower sick leave costs for females seem contradictory. An explanation for the unexpected low productivity losses for females could be the difference in average working hours between the sexes: females work in general less hours than males in the Netherlands [11]. The multivariate analysis, however, corrects for weekly working hours and despite that females still generated fewer productivity losses than males. Therefore, the male–female pay gap, which is reflected in the age and sex-dependent average hourly gross wage, is likely to be responsible for the lower average sick leave costs of females. Considering the intent of the European Union to close the male–female pay gap, and because differences in wage due to sex decrease every year, we recommend policymakers and employers to target their interventions to prevent and reduce sick leave among females and therefore even interventions with a cost-minimizing strategy should focus on females to support the more sustainable policy [32].

### Generalizability

When comparing the characteristics of our study sample with those of the Dutch workforce the distribution of OA by age groups and the distribution of working hours are relatively similar [12]. However, the distribution of OA by sex in our sample differed from the overall Dutch population where females make up the majority of knee and hip OA patients: on average 60% [12]. This is not the case in our sample, especially for knee OA, where 60% of the workers on sick leave are male. Research from France observed the same phenomenon in the French workforce, where the authors argued that, as the workforce consists of relatively more males, the prevalence of OA in the workforce is consequently higher among males [33]. The Dutch workforce consists of 57% of males and could therefore explain the male–female ratio for knee and hip OA in our sample. Potentially, traditional differences in the types of occupations and levels of physical demands in the work undertaken by males and females could also explain why knee OA is more prevalent among males in the workforce. In the Netherlands, traditional masculine occupations like industrial work and construction are male-dominated sectors (83% male), and involve a lot of repetitive kneeling and squatting, in combination with heavy lifting, which are known risk factors for knee OA [34]. However, whether the higher percentage of males in the working population and/or traditional differences in types of occupation are indeed responsible for the distribution of OA by sex, could not be verified in our dataset since data on the occupational sectors were not available. This assumption should be tested, despite the magnitude of the our dataset with 1,2 million workers (14% of the Dutch working population) including 13,000 small, medium and large companies appears representative for the nation. Therefore, more research is needed to better understand the sex differences found for the prevalence of knee- and hip OA in the working population.

### Methodological strengths and limitations

A strength of our study is that our database represents physician-diagnosed sick leave episodes of knee and hip OA among a sample of 1.2 million workers, supporting the generalizability of our results for the Netherlands. We took different perspectives and methods of cost calculation into account to assess the robustness of our findings in order to increase comparability across countries and satisfy the needs of both policymakers and employers by estimating sick leave costs from a societal perspective as well as from an employer’s perspective. Furthermore, the costs and sick leave days were corrected for the recovery percentage which increases the accuracy of our estimates.

However, it must be noted that this study had some limitations. There is a possibility that our estimated costs are an underestimation of the actual sick leave-related costs. We based cost calculations on the legally required minimum payment a Dutch employer must make to their absent worker. This is 70% of the worker's wage for the first two years of sick leave [15]. However, this estimation is quite conservative because employers have the liberty to pay the absent worker more and most collective labor agreements prescribe 100% salary payment in the first year of sick leave and 70% in the second year. Therefore, costs could certainly be higher than estimated in this study but are, most likely, not lower since this would violate Dutch legislation. Furthermore, excluding episodes that continued into 2020 could have led to an underestimation of costs per episode. However, we expect the underestimation to be small because this is only true for episodes starting in 2018, with only 26 participants being excluded for this reason. Episodes that started in the other years did not have this limitation. Moreover, workers with less than 4 weekly working hours were excluded from our analyses and self-employed workers were not represented in our dataset. Not considering these workers could have caused selection bias, which could have led to different results of the average costs per episode.

Literature suggests the possibility of other variables influencing the prevalence and severity of OA and therefore sick leave episodes, like obesity, occupational factors, and other comorbidities [35, 36]. These variables could have altered the results regarding for instance the specific workers at risks. Therefore, further research on this topic is recommended. As said, there was no possibility to collect these additional data from our study participants, because the data in the pre-existing dataset were already anonymized.

## Conclusion

Annual sick leave costs due to knee and hip OA in the Dutch workforce are substantial, with estimated costs of €40 million and costs being approximately twice as high for knee compared to hip OA. Average costs per sick leave episode are also considerable, €15.550 for knee OA and €12.482 for hip OA, and indicate the magnitude of financial burden of osteoarthritis for society. As costs are particularly high among male workers and workers with a higher number of weekly working hours, such groups could be focused on in attempts to reduce the societal burden.

## Supplementary Information

**Additional file 1.**

**Additional file 2.**

**Additional file 3.**

**Additional file 4.**

## Data Availability

Data used for this study are owned by Arbo Unie. Data can be obtained from Arbo Unie upon request.

## Abbreviations

OA: Osteoarthritis
95%CI: 95% Confidence intervals
FCA: Friction cost approach
HCA: Human capital approach
