# Supplementary material for: The economic burden of knee and hip osteoarthritis: absenteeism and costs in the Dutch workforce
Source: BMC Musculoskelet Disord. 2022 Apr 18;23:364. doi: 10.1186/s12891-022-05306-9 (PMC9017043; doi:10.1186/s12891-022-05306-9)
Supplement: Supplementary file 1 — Additional file 1. [file 12891_2022_5306_MOESM1_ESM.docx]

**Supplementary file 1.** Age and sex-dependent average national gross wage per hour in the Netherlands.

|  | 2013 | 2014 | 2015 | 2016 | 2017 | 2018 | 2019 |
| --- | --- | --- | --- | --- | --- | --- | --- |
| Male | | | | | | | |
| 15 – 20 | € 6.09 | € 6.00 | € 6.05 | € 6.01 | € 6.24 | € 6.69 | € 7.08 |
| 20 – 25 | € 11.63 | € 11.49 | € 11.64 | € 11.69 | € 12.00 | € 12.63 | € 13.13 |
| 25 – 30 | € 16.21 | € 16.08 | € 16.30 | € 16.36 | € 16.56 | € 17.15 | € 17.72 |
| 30 – 35 | € 20.08 | € 20.03 | € 20.34 | € 20.34 | € 20.47 | € 21.04 | € 21.63 |
| 35 – 40 | € 23.50 | € 23.47 | € 23.89 | € 23.93 | € 24.03 | € 24.54 | € 25.05 |
| 40 – 45 | € 25.90 | € 26.03 | € 26.67 | € 26.71 | € 26.79 | € 27.25 | € 27.70 |
| 45 – 50 | € 27.11 | € 27.33 | € 28.11 | € 28.21 | € 28.38 | € 28.89 | € 29.38 |
| 50 – 55 | € 27.67 | € 27.87 | € 28.68 | € 28.78 | € 28.95 | € 29.52 | € 30.04 |
| 55 – 60 | € 27.69 | € 27.87 | € 28.75 | € 28.93 | € 28.99 | € 29.51 | € 30.01 |
| 60 – 65 | € 27.38 | € 27.25 | € 27.97 | € 28.09 | € 28.20 | € 28.64 | € 29.13 |
| 65 – 75 | € 22.21 | € 22.59 | € 23.67 | € 24.36 | € 24.89 | € 25.45 | € 25.96 |
| ≥ 75 | € 16.43 | € 16.36 | € 16.99 | € 17.39 | € 18.24 | € 19.11 | € 19.70 |
| Female | | | | | | | |
| 15 – 20 | € 5.79 | € 5.81 | € 5.86 | € 5.68 | € 5.94 | € 6.30 | € 6.66 |
| 20 – 25 | € 11.53 | € 11.41 | € 11.51 | € 11.51 | € 11.84 | € 12.45 | € 12.97 |
| 25 – 30 | € 16.45 | € 16.38 | € 16.60 | € 16.63 | € 16.78 | € 17.29 | € 17.85 |
| 30 – 35 | € 19.72 | € 19.78 | € 20.15 | € 20.23 | € 20.35 | € 20.83 | € 21.33 |
| 35 – 40 | € 21.68 | € 21.93 | € 22.48 | € 22.65 | € 22.81 | € 23.30 | € 23.78 |
| 40 – 45 | € 21.99 | € 22.34 | € 23.08 | € 23.42 | € 23.64 | € 24.16 | € 24.69 |
| 45 – 50 | € 21.52 | € 21.89 | € 22.64 | € 23.01 | € 23.29 | € 23.91 | € 24.42 |
| 50 – 55 | € 21.42 | € 21.67 | € 22.33 | € 22.62 | € 22.83 | € 23.36 | € 23.88 |
| 55 – 60 | € 21.56 | € 21.65 | € 22.28 | € 22.52 | € 22.73 | € 23.21 | € 23.64 |
| 60 – 65 | € 21.32 | € 21.39 | € 21.98 | € 22.25 | € 22.54 | € 23.01 | € 23.45 |
| 65 – 75 | € 17.50 | € 17.65 | € 18.67 | € 19.63 | € 20.41 | € 21.13 | € 21.79 |
| ≥ 75 | € 12.51 | € 12.98 | € 13.50 | € 14.05 | € 15.44 | € 15.95 | € 16.41 |
