# Supplementary material for: The economic burden of knee and hip osteoarthritis: absenteeism and costs in the Dutch workforce
Source: BMC Musculoskelet Disord. 2022 Apr 18;23:364. doi: 10.1186/s12891-022-05306-9 (PMC9017043; doi:10.1186/s12891-022-05306-9)
Supplement: Supplementary file 2 — Additional file 2. [file 12891_2022_5306_MOESM2_ESM.docx]

**Supplementary file 2.** Calculation friction period

Vacancies in the Netherlands

|  | 2013 | 2014 | 2015 | 2016 | 2017 | 2018 | 2019 |
| --- | --- | --- | --- | --- | --- | --- | --- |
| Outstanding vacancies (x1000) | 95.1 | 108.2 | 129.7 | 155.9 | 200.6 | 248.3 | 281.1 |
| Filled vacancies (x1000) | 624 | 689 | 800 | 887 | 999 | 1146 | 1232 |

Friction period

$$Friction period=365 /\frac{Filled vacancies}{Outstanding vacancies}+4 weeks$$

Calculated friction period per year

|  | 2013 | 2014 | 2015 | 2016 | 2017 | 2018 | 2019 |
| --- | --- | --- | --- | --- | --- | --- | --- |
| Vacancy period (days) | 56 | 58 | 60 | 65 | 74 | 80 | 84 |
| Friction period (days) | 84 | 86 | 88 | 93 | 102 | 108 | 112 |
| Friction period (weeks) | 12.0 | 12.2 | 12.5 | 13.2 | 14.5 | 15.3 | 15.9 |
