# Supplementary material for: The economic burden of knee and hip osteoarthritis: absenteeism and costs in the Dutch workforce
Source: BMC Musculoskelet Disord. 2022 Apr 18;23:364. doi: 10.1186/s12891-022-05306-9 (PMC9017043; doi:10.1186/s12891-022-05306-9)
Supplement: Supplementary file 3 — Additional file 3. [file 12891_2022_5306_MOESM3_ESM.docx]

**Supplementary file 3.** Sick leave days and absenteeism costs per episode of knee osteoarthritis and per episode of hip osteoarthritis Human Capital Approach and an employer´s perspective

Sick leave days and absenteeism costs per episode of knee osteoarthritis, calculated using the Human Capital Approach and an employer´s perspective. Univariate and multivariate (adjusting for all other factors) regression models are shown, depicting subgroup differences. Here beta (B) with 95 % confidence intervals (95% CI) are reported.

|  | | Sick leave days | | Costs (€) | | Univariate model | | | Multivariate model | | |
| --- | --- | --- | --- | --- | --- | --- | --- | --- | --- | --- | --- |
|  |  | **Mean** | **95% CI** | **Mean** | **95% CI** | **B** | **95% CI** | **p-value** | **B** | **95% CI** | **p-value** |
| Overall | | 186 | 176 – 196 | 15,550 | 14,574 – 16,541 |  | | |  | | |
| Sex | Male | 187 | 174 – 202 | 19,032 | 17,585 – 20,532 | Reference | |  |  |  |  |
|  | Female | 183 | 168 – 200 | 10,187 | 9,249 – 11,180 | -8796 | -10536 – -7072 | 0.000 | -4360 | -6411 – -2251 | 0.000 |
| Age | <45 | 193 | 136 – 256 | 13,787 | 9,499 – 18,667 | Reference | |  |  |  |  |
|  | 45-49 | 164 | 133 – 201 | 13,897 | 11,251 – 16,830 | 110 | -5410 – 5517 | 0.969 | 466 | -4905 – 5631 | 0.871 |
|  | 50-54 | 190 | 164 – 219 | 16,782 | 14,088 – 19,621 | 2994 | -3075 – 8733 | 0.311 | 3415 | -1987 – 8804 | 0.235 |
|  | 55-59 | 190 | 171 – 211 | 15,701 | 13,854 – 17,668 | 1914 | -3379 – 6861 | 0.480 | 2876 | -2103 – 7865 | 0.274 |
|  | 60-64 | 187 | 170 – 205 | 15,958 | 14,315 – 17,736 | 2106 | -2975 – 7105 | 0.436 | 2348 | -2486 – 7036 | 0.367 |
|  | ≥65 | 167 | 139 – 198 | 12,541 | 9,595 – 15,866 | -1246 | -6798 – 4323 | 0.679 | -671 | -5816 – 4414 | 0.816 |
| Hours | <20 | 178 | 152 – 204 | 5,637 | 4,763 – 6,607 | Reference | |  |  |  |  |
|  | 20-24 | 218 | 178 – 262 | 11,672 | 9,467 – 14,148 | 6035 | 3509 – 8441 | 0.000 | 5892 | 3415 – 8436 | 0.000 |
|  | 25-29 | 202 | 163 – 245 | 13,432 | 10,788 – 16,384 | 7795 | 5018 – 10715 | 0.000 | 7702 | 4790 – 10639 | 0.000 |
|  | 30-34 | 182 | 152 - 214 | 14,475 | 12,061 – 16,976 | 8658 | 6033 – 11313 | 0.000 | 7808 | 5147 – 10511 | 0.000 |
|  | 35-39 | 158 | 143 – 174 | 15,769 | 14,241 – 17,447 | 10132 | 8114 – 12128 | 0.000 | 7229 | 4982 – 9499 | 0.000 |
|  | ≥40 | 204 | 184 – 224 | 22,715 | 20,502 – 25,000 | 17078 | 14489 – 19736 | 0.000 | 13922 | 11136 – 16713 | 0.000 |
| Sick leave episode | 1^st^ | 186 | 176 – 198 | 15,332 | 14,314 – 16,415 | Reference | |  |  |  |  |
|  | >1 | 180 | 149 – 214 | 17,726 | 14,275 – 21,630 | 2418 | -1416 – 6201 | 0.221 | 303 | -3260 – 3747 | 0.870 |

Sick leave days and absenteeism costs per episode of hip osteoarthritis, calculated using the Human Capital Approach and an employer’s perspective. Univariate and multivariate (adjusting for all other factors) regression models are shown, depicting subgroup differences. Here beta (B) with 95 % confidence intervals (95% CI) are reported.

|  | | Sick leave days | | Costs (€) | | Univariate model | | | Multivariate model | | |
| --- | --- | --- | --- | --- | --- | --- | --- | --- | --- | --- | --- |
|  |  | **Mean** | **95% CI** | **Mean** | **95% CI** | **B** | **95% CI** | **p-value** | **B** | **95% CI** | **p-value** |
| Overall | | 159 | 148 – 171 | 12,482 | 11,469 – 13,566 |  | | |  | | |
| Sex | Male | 147 | 133 – 163 | 14,939 | 13,349 – 16,645 | Reference | | |  | | |
|  | Female | 173 | 157 – 190 | 9,822 | 8,792 – 10,917 | -5116 | -7125 – -3051 | 0.000 | -1875 | -4151 – 370 | 0.104 |
| Age | <45 | 207 | 149 – 274 | 13,800 | 9,544 – 18,888 | Reference | | |  | | |
|  | 45-49 | 143 | 118 – 170 | 10,795 | 8,762 – 13,142 | -3005 | -8033 – 1998 | 0.275 | -3255 | -8212 – 1659 | 0.221 |
|  | 50-54 | 181 | 148 – 218 | 15,216 | 12,252 – 18,719 | 1416 | -4112 – 7202 | 0.635 | 1013 | -4529 – 6454 | 0.736 |
|  | 55-59 | 151 | 132 – 173 | 12,151 | 10,334 – 14,207 | -1649 | -6520 – 3177 | 0.550 | -1270 | -6031 – 3438 | 0.632 |
|  | 60-64 | 158 | 142 – 175 | 12,320 | 10,920 – 13,839 | -1480 | -6139 – 3262 | 0.573 | -1094 | -5776 – 3579 | 0.669 |
|  | ≥65 | 104 | 83 – 126 | 6,965 | 4,887 – 9,169 | -6836 | -11800 – -1965 | 0.006 | -6627 | -11815 – -1493 | 0.008 |
| Hours | <20 | 166 | 138 – 198 | 4,826 | 3,983 – 5,818 | Reference | | |  | | |
|  | 20-24 | 206 | 171 – 246 | 10,672 | 8,789 – 12,837 | 5846 | 3592 – 8142 | 0.000 | 6053 | 3835 – 8193 | 0.000 |
|  | 25-29 | 158 | 127 – 192 | 10,575 | 8,422 – 13,118 | 5749 | 3195 – 8359 | 0.000 | 5140 | 2624 – 7588 | 0.000 |
|  | 30-34 | 140 | 113 – 170 | 11,002 | 8,855 – 13,538 | 6176 | 3662 – 8818 | 0.000 | 5435 | 2696 – 8245 | 0.000 |
|  | 35-39 | 151 | 133 – 173 | 14,788 | 12,935 – 16,910 | 9961 | 7621 – 12279 | 0.000 | 8622 | 6215 – 11063 | 0.000 |
|  | ≥40 | 151 | 130 – 175 | 16,388 | 14,033 – 19,077 | 11562 | 8729 – 14473 | 0.000 | 10163 | 7296 – 12978 | 0.000 |
| Sick leave episode | 1^st^ | 158 | 147 – 170 | 12,358 | 11,422 – 13,406 | Reference | | |  | | |
|  | >1 | 174 | 136 – 217 | 13,791 | 10,163 – 18,025 | 1433 | -2908 – 5716 | 0.539 | 1791 | -2281 – 5883 | 0.409 |
