# Supplementary material for: The economic burden of knee and hip osteoarthritis: absenteeism and costs in the Dutch workforce
Source: BMC Musculoskelet Disord. 2022 Apr 18;23:364. doi: 10.1186/s12891-022-05306-9 (PMC9017043; doi:10.1186/s12891-022-05306-9)
Supplement: Supplementary file 4 — Additional file 4. [file 12891_2022_5306_MOESM4_ESM.docx]

**Supplementary file 4.** Sick leave days and absenteeism costs per episode of knee osteoarthritis and hip osteoarthritis, calculated using the Friction Cost Approach and an employer´s perspective. Univariate and multivariate (adjusting for all other factors) regression models are shown, depicting subgroup differences. Here beta (B) with 95 % confidence intervals (95% CI) are reported.

| Knee Osteoarthritis, Friction Cost Approach, employer’s perspective | | | | | | | | | | | |
| --- | --- | --- | --- | --- | --- | --- | --- | --- | --- | --- | --- |
|  | | **Sick leave days** | | **Costs (€)** | | **Univariate model** | | | **Multivariate model** | | |
|  |  | **Mean** | **95% CI** | **Mean** | **95% CI** | **B** | **95% CI** | **p-value** | **B** | **95% CI** | **p-value** |
| Overall | | 81 | 79 – 82 | 6,736 | 6,528 – 6,936 |  | | |  | | |
| Sex | Male | 80 | 78 – 83 | 8,142 | 7,896 – 8,306 | Reference | |  |  |  |  |
|  | Female | 81 | 79 – 84 | 4,571 | 4,331 – 4,799 | -3561 | -3889 – -3261 | 0.000 | -1325 | -1638 – -1039 | 0.000 |
| Age | <45 | 73 | 63 – 83 | 5,619 | 4,446 – 6,774 | Reference | |  |  |  |  |
|  | 45-49 | 74 | 69 – 80 | 6,486 | 5,751 – 7,235 | 868 | -430 – 2201 | 0.204 | 906 | -135 – 1973 | 0.088 |
|  | 50-54 | 78 | 73 – 82 | 6,667 | 6,182 – 7,145 | 1049 | -144 – 2226 | 0.107 | 1147 | 162 – 2164 | 0.025 |
|  | 55-59 | 80 | 77 – 83 | 6,625 | 6,247 – 6,994 | 1006 | -131 – 2194 | 0.096 | 1270 | 340 – 2270 | 0.007 |
|  | 60-64 | 84 | 82 – 86 | 7,157 | 6,814 – 7,488 | 1526 | 377 – 2680 | 0.009 | 1513 | 608 – 2469 | 0.000 |
|  | ≥65 | 87 | 79 – 93 | 6,079 | 5,114 – 6,986 | 460 | -1001 – 1989 | 0.540 | 764 | -345 – 1878 | 0.182 |
| Hours | <20 | 79 | 75 – 83 | 2,506 | 2,298 – 2,709 | Reference | |  |  |  |  |
|  | 20-24 | 85 | 81 – 91 | 4,590 | 4,297 – 4,869 | 2976 | 2487 – 3471 | 0.000 | 2077 | 1740 – 2406 | 0.000 |
|  | 25-29 | 86 | 81 – 91 | 5,731 | 5,302 – 6,125 | 4606 | 3974 – 5197 | 0.000 | 3191 | 2801 – 3585 | 0.000 |
|  | 30-34 | 80 | 75 – 84 | 6,242 | 5,843 – 6,631 | 5307 | 4675 – 5931 | 0.000 | 3452 | 3012 – 3882 | 0.000 |
|  | 35-39 | 79 | 76 – 82 | 7,883 | 7,561 – 8,186 | 7681 | 7182 – 8161 | 0.000 | 4482 | 4093 – 4833 | 0.000 |
|  | ≥40 | 81 | 78 – 84 | 8,998 | 8,651 – 9,323 | 9273 | 8714 – 9857 | 0.000 | 5518 | 5087 – 5963 | 0.000 |
| Sick leave episode | 1^st^ | 81 | 79 – 82 | 6,624 | 6,403 – 6,845 | Reference | |  |  |  |  |
|  | >1 | 83 | 77 – 88 | 7,854 | 7,153 – 8,552 | 1233 | 496 – 1950 | 0.001 | 394 | -118 – 917 | 0.132 |
| Hip osteoarthritis, Friction Cost Approach, employer’s perspective | | | | | | | | | | | |
|  | | **Sick leave days** | | **Costs (€)** | | **Univariate model** | | | **Multivariate model** | | |
|  |  | **Mean** | **95% CI** | **Mean** | **95% CI** | **B** | **95% CI** | **p-value** | **B** | **95% CI** | **p-value** |
| Overall | | 82 | 80 – 83 | 6,462 | 6,209 – 6,721 |  | | |  | | |
| Sex | Male | 78 | 75 – 80 | 7,818 | 7,471 – 8161 | Reference | | |  | | |
|  | Female | 86 | 84 – 88 | 4,994 | 4,737 – 5,254 | -2824 | -3261 – -2383 | 0.000 | -887 | -1284 – -490 | 0.000 |
| Age | <45 | 81 | 74 – 88 | 5,610 | 4,682 – 6,541 | Reference | | |  | | |
|  | 45-49 | 77 | 70 – 84 | 6,142 | 5,288 – 7,010 | 532 | -715 – 1805 | 0.407 | 589 | -398 – 1559 | 0.244 |
|  | 50-54 | 82 | 78 – 86 | 7,059 | 6,452 – 7,671 | 1449 | 365 – 2530 | 0.010 | 1291 | 461 – 2146 | 0.002 |
|  | 55-59 | 80 | 76 – 84 | 6,368 | 5,873 – 6,863 | 758 | -261 – 1783 | 0.151 | 952 | 121 – 1784 | 0.023 |
|  | 60-64 | 84 | 81 – 86 | 6,605 | 6,180 – 7,024 | 995 | 4 – 1981 | 0.051 | 1203 | 425 – 1987 | 0.002 |
|  | ≥65 | 82 | 68 – 93 | 5,345 | 3,960 – 6,699 | -265 | -1833 – 1246 | 0.741 | 321 | -886 – 1534 | 0.617 |
| Hours | <20 | 84 | 79 – 88 | 2,498 | 2,233 – 2,761 | Reference | | |  | | |
|  | 20-24 | 88 | 85 – 91 | 4,559 | 4,328 – 4,774 | 2945 | 2418 – 3448 | 0.000 | 2154 | 1800 – 2500 | 0.000 |
|  | 25-29 | 82 | 77 – 87 | 5,496 | 5,039 – 5,959 | 4283 | 3571 – 5009 | 0.000 | 2886 | 2393 – 3378 | 0.000 |
|  | 30-34 | 80 | 73 – 86 | 6,243 | 5,674 – 6,784 | 5350 | 4510 – 6166 | 0.000 | 3463 | 2865 – 4064 | 0.000 |
|  | 35-39 | 81 | 77 – 84 | 7,871 | 7,503 – 8,238 | 7676 | 7043 – 8269 | 0.000 | 4851 | 4382 – 5323 | 0.000 |
|  | ≥40 | 79 | 75 – 83 | 8,457 | 7,997 – 8,908 | 8514 | 7749 – 9282 | 0.000 | 5407 | 4834 – 5975 | 0.000 |
| Sick leave episode | 1^st^ | 82 | 80 – 84 | 6,495 | 6,229 – 6,743 | Reference | | |  | | |
|  | >1 | 79 | 71 – 85 | 6,122 | 5,269 – 7,010 | -373 | -1253 – 526 | 0.425 | -353 | -993 – 300 | 0.301 |
